# A Systems Biology-Based Classifier for Hepatocellular Carcinoma Diagnosis

**DOI:** 10.1371/journal.pone.0022426

**Published:** 2011-07-28

**Authors:** Yanqiong Zhang, Shaochuang Wang, Dong Li, Jiyang Zhnag, Dianhua Gu, Yunping Zhu, Fuchu He

**Affiliations:** 1 Institute of Basic Medical Sciences, Chinese Academy of Medical Sciences & Peking Union Medical College, Beijing, People's Republic of China; 2 State Key Laboratory of Proteomics, Beijing Proteome Research Center, Beijing Institute of Radiation Medicine, Beijing, People's Republic of China; 3 Department of Hepatobiliary and Pancreatic Surgery, Nanjing Medical University Affiliated Huai'an 1st People's Hospital, Huai'an, Jiangsu, People's Republic of China; 4 Department of Automatic Control, College of Mechatronics and Automation, National University of Defense Technology, Changsha, Hunan, People's Republic of China; Kyushu Institute of Technology, Japan

## Abstract

**Aim:**

The diagnosis of hepatocellular carcinoma (HCC) in the early stage is crucial to the application of curative treatments which are the only hope for increasing the life expectancy of patients. Recently, several large-scale studies have shed light on this problem through analysis of gene expression profiles to identify markers correlated with HCC progression. However, those marker sets shared few genes in common and were poorly validated using independent data. Therefore, we developed a systems biology based classifier by combining the differential gene expression with topological features of human protein interaction networks to enhance the ability of HCC diagnosis.

**Methods and Results:**

In the Oncomine platform, genes differentially expressed in HCC tissues relative to their corresponding normal tissues were filtered by a corrected Q value cut-off and Concept filters. The identified genes that are common to different microarray datasets were chosen as the candidate markers. Then, their networks were analyzed by GeneGO Meta-Core software and the hub genes were chosen. After that, an HCC diagnostic classifier was constructed by Partial Least Squares modeling based on the microarray gene expression data of the hub genes. Validations of diagnostic performance showed that this classifier had high predictive accuracy (85.88∼92.71%) and area under ROC curve (approximating 1.0), and that the network topological features integrated into this classifier contribute greatly to improving the predictive performance. Furthermore, it has been demonstrated that this modeling strategy is not only applicable to HCC, but also to other cancers.

**Conclusion:**

Our analysis suggests that the systems biology-based classifier that combines the differential gene expression and topological features of human protein interaction network may enhance the diagnostic performance of HCC classifier.

## Introduction

Hepatocellular carcinoma (HCC) is one of the most common malignant tumors with an increasing incidence worldwide. The resistance of HCC to existing treatments and the lack of biomarkers for early detection make it one of the most hard-to-treat cancers. High-risk patients with HCC are closely followed up and increasing numbers of small equivocal lesions, which are widely recognized as dysplastic nodules or early-stage HCC, lack typical imaging and histology of ordinary HCC and do not show elevated serum markers, such as alpha-fetoprotein (AFP) and PIVKA-II [1]–[2]. Given the importance of early-stage diagnosis to the application of curative treatments which are the only hope for increasing the life expectancy of patients with HCC, the development of effective systems which can predict the occurrence of this neoplasm is much needed.

Several attempts have been made to predict the occurrence and prognosis of HCC based on single or multiple clinicopathologic features such as the severity of the liver function, age, tumor size, grade, microvascular invasion, portal vein thrombosis, and the presence of microsatellite regions [3]–[4]. However, their clinical applicability is worthy of further large-scale validations. Recent studies on gene expression profiles could successfully predict the occurrence, progression, or survival of cancers [5]–[6], but the lack of consistency of these microarray-based predictors generated from the heterogeneity of the patient cohorts and the difference in microarray platforms remain one of the major obstacles to their clinical use, making it necessary to identify a reliable and consistent predictor that is robust enough to overcome the variabilities induced by different platforms or different patient cohorts.

There have been several approaches to this problem from different perspectives. One approach performs a gene pathway-based analysis, which identifies biological pathways by scoring the coherency of expression changes among their member genes based on microarray data [7]. Such a method allows biologists to incorporate previously accumulated biological knowledge in the analysis and make a more biology-driven analysis of microarray data, which can help identify interpretable discriminative signatures that gains insight into tumor biology and potential therapeutic targets. In addition, this method enables the identification of moderately differentially expressed but functionally important genes, which are missed in gene expression clustering. A second approach is a protein interaction network-based method, which utilizes a recently available protein-protein interaction network to identify sub-networks based on coherent expression patterns of their genes [8]. A sub-network refers to a smaller or more focused network within a large protein interaction network [9]. Both methods efficiently utilize co-expression information embedded within the microarray gene expression data. However, the problem with both methods is that each gene set or sub-network identified includes too many genes, which greatly limits their clinical application.

Lu et al. [10] demonstrated that hubs of biological network have significantly different biological functions compared with peripheral nodes based on Gene Ontology classification, and that biological understanding of experimental asthma is enhanced by combining information including levels of change in gene expression plus topological criteria from the analysis of interaction networks. We hypothesized that developing a systems biology-based approach by combining differential gene expression and topological characteristics of human protein interaction networks could improve the diagnostic performance of HCC classifier.

## Materials and Methods

**Figure 1:**
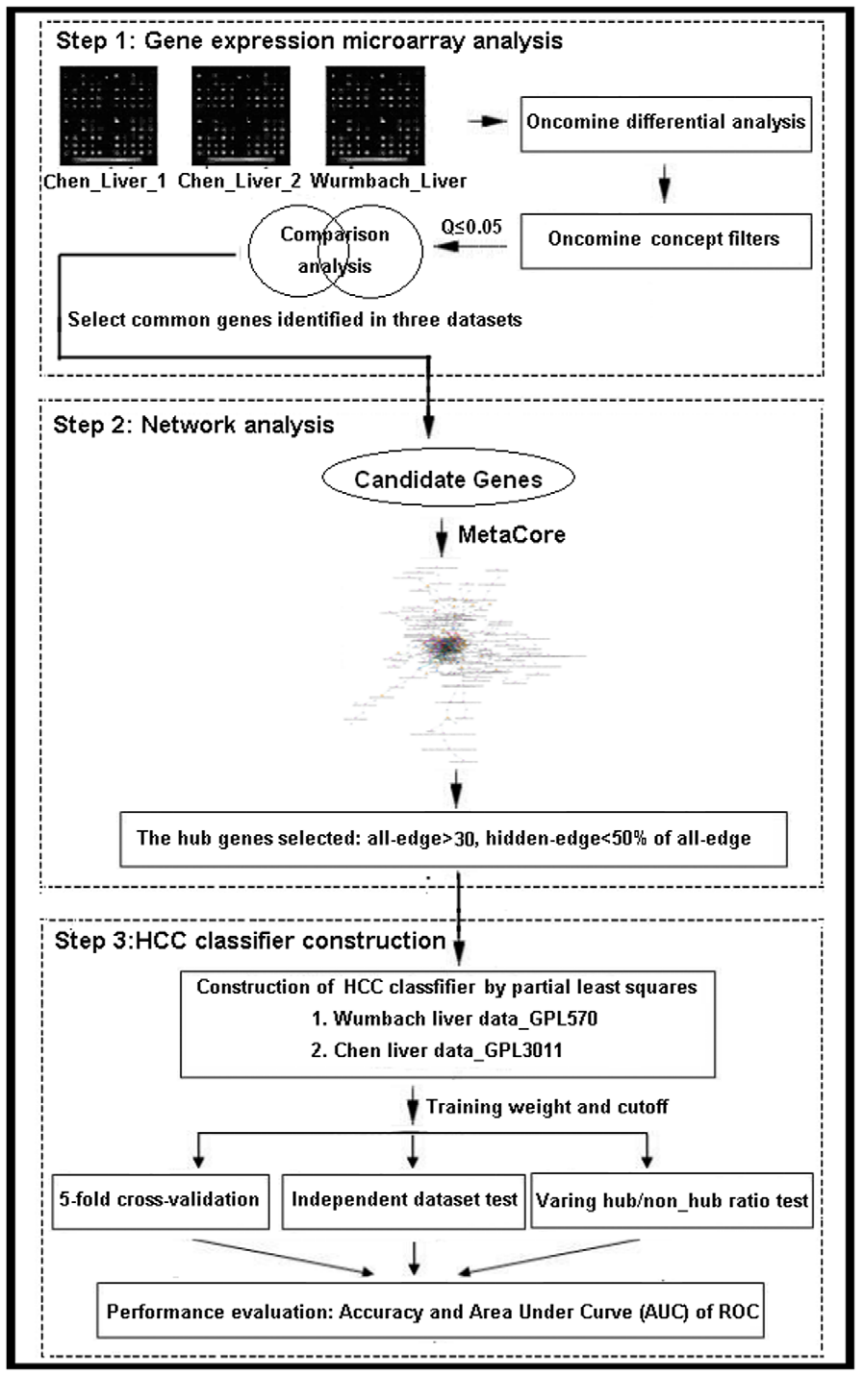
A schematic diagram of this novel systems biology-based gene expression classifier for HCC diagnosis. First, the genes differentially expressed in HCC tissues relative to their corresponding non-tumor tissues were filtered by a corrected Q value cut-off and Concept filters in the Oncomine platform. The identified genes that are common among different microarray datasets were chosen as the candidate genes. Then, their networks were analyzed by GeneGO Meta-Core software and the hub genes were chosen. After that, HCC diagnostic classifier was constructed by PLS modeling based on the microarray gene expression data of the hub genes. Finally, the diagnostic performance of this classifier was evaluated by predictive accuracy and area under ROC curve.

The technical strategy of this study was shown in Figure 1.

### Gene expression microarray analysis

The data mining strategy for selecting marker genes for our classifier is based on a published methodology exploring the cancer microarray platform, Oncomine [11] (16 SEP 2008 ONCOMINE DATA RELEASE HIGHLIGHTS, https://www.oncomine.org), which was chosen because it is a public cancer microarray platform incorporating 392 independent microarray datasets, totaling more than 28,880 microarray experiments and spanning 41 cancer types. It unifies a large compendium of other published cancer microarray data, including Gene Expression Omnibus (GEO) [12] and Stanford Microarray Database (SMD) [13], and uniquely provides differential expression analyses comparing most major types of cancer with their respective normal tissues. For example, to identify potentially important genes in a particular cancer, users can perform a “cancer vs. normal” analysis for a given cancer type and those genes that are differentially expressed in cancer relative to its normal tissue can be retrieved as a list. Each differentially expressed gene in the list can then be assessed by the Student t test to calculate the P or Q values (false discovery rate) [14], mean expression values (mean 1, mean 2), and the normalized Student t value. In addition, Oncomine is integrated with the Concept filter, which allows users to identify genes with certain biological processes or mutation types.

#### Public expression datasets

Hepatocellular carcinoma was used in the <profile search> function in the Oncomine database to find the available microarray datasets related to the specific cancer type. The analysis type <cancer vs. non-tumor> was then applied to filter those microarray datasets exploring cancer relative to its non-tumor tissue. Three publicly available datasets of gene expression profiles were chosen in this study, including Chen_Liver_1 [15] (Non-tumor Liver vs. Hepatocellular Carcinoma), Chen_Liver_2 [15] (Non-tumor Liver vs. Hepatocellular Carcinoma), and Wurmbach_Liver [16] (Non-tumor Liver vs. Hepatocellular Carcinoma). The detailed information about the datasets is described in Raw Data S1, S2, S3, S4, S5 and Table S1.

#### Gene selection procedure

Concept filters in the Oncomine database were used to identify known oncogenes differentially expressed in HCC. Specifically, differentially expressed genes associated with the following Concept filter terms were searched: <CAN Genes>, <The Cancer Gene Census>, <Catalogue of Somatic Mutations in Cancer (COSMIC)>, <Drug target-experimental> and <Drug target-FDA>. Next, a corrected false discovery rate Q value threshold (Q≤0.05) was used to filter and retrieve those differentially expressed genes with a high confidence. Then, the differentially expressed genes identified throughout the three microarray gene expression datasets were selected as candidate genes for further network analysis.

### Network analysis

The network representation was generated using GeneGO Meta-Core software (Encinitas, CA). The software interconnected all candidate genes according to published literature-based annotations. Only direct connections between the identified genes were considered. Major hubs were defined as those with more than thirty connections and <50% of edges hidden within the network. The hub genes were selected as the components of HCC classifier.

### Systems biology-based HCC classifier construction

#### Datasets

To demonstrate this novel classifier, two publicly available datasets of gene expression profiles were used in this study, including Chen_Liver [15] (29 HCC samples and 17 non-tumor liver samples) and Wurmbach_Liver [16] (35 HCC samples and 27 non-tumor liver samples) datasets. These datasets were randomly separated into the training and test datasets for 100 times. The detailed information about the datasets is described in Raw Data S1, S2, S3, S4, S5.

#### Parial Least Squares classifier

Parial Least Squares (PLS), which can extract effective information form a larger number of predictors, was used to construct the systems biology-based HCC classifier. To describe this method, some notations are required. Let 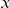 be 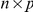 matrix of 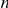 samples and 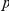 hub genes. Also, let 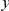 denote the 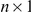 vector of response values, such as the indicator of HCC or non-tumor liver tissues. The objective criterion for constructing components in PLS is to sequentially maximize the covariance between the response variable and a linear combination of the predictors. The components are constructed to maximize the objective criterion based on the sample covariance between 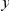 and 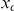. Thus, we find the weight vector 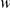 satisfying the following objective criterion,
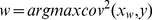
(1)Next, a training dataset was used to calculate weight coefficients of different hub genes in PLS model. The hub genes in PLS model are denoted as:
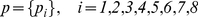
(2)The score of the PLS model for each sample is defined as:
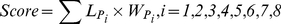
(3)Where 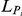 refers to the expression level of hub gene 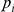 in each sample.

Then, the training dataset was used to input the PLS model so as to calculate the threshold value 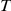 of score by selecting the cutoff value on which the Area under Receiver Operating Characteristic (ROC) Curve (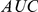) was the biggest. Finally, the PLS classifier decides: if 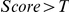, the sample can be predicted as HCC tissues.

### Performance evaluation

The overall performance of HCC classifier was evaluated by two distinct approaches: 5-fold cross-validation test and independent dataset test. The overall predictive accuracy (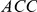) and 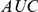 were used to measure the prediction performance of our method. ROC Curve can show the efficacy of one test by presenting both sensitivity and specificity for different cutoff points [17]. Sensitivity and specificity can measure the ability of a test to identify true positives and false ones in a dataset.
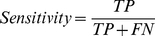
(4)

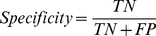
(5)

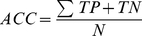
(6)where 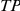, 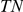, 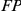, 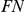 respectively refer to the number of true positive, true negative, false positive and false negative result components in a test, while 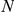 refers to the total number of predicted samples.

The ROC curves are plotted and smoothed by SPSS software with the sensitivity on the 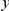 axis and 1-Specificity on the 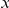 axis.

In the 5-fold cross-validation test, the dataset was randomly divided into 5 sets, four of which were used to train the parameters of the predictive algorithm. The predictive accuracy of the algorithm was then evaluated by the remaining set, and this procedure was repeated five times before sensitivity and specificity against different parameters across five test datasets are calculated for the ROC curve.

### Validation of MAPK1 and NCOA2 hubs

#### Patients and tumor tissue specimens

The study was approved by the Research Ethics Committee of Affiliated Huai'an 1^st^ People's Hospital, Nanjing Medical University Huai'an, Jiangsu, P.R.China. Written Informed Consent was obtained from each of the patients. All specimens were handled and made anonymous according to the ethical and legal standards.

Thirty matched HCC and paracarcinomatous liver tissue (PCLT) specimens were obtained from 30 patients who underwent hepatectomy at the Department of Hepatobiliary & Pancreatic Surgery at this hospital between 2007 and 2009. These patients included 22 males and 8 females with a median age of 52.4 years (range, 30∼76 years). No patients who had undergone previous therapy or non-curative surgery were included. The clinicopathologic variables, such as tumor size, etiological factors, underlying disease, portal vein infiltration, Edmondson-Steiner grade, TNM stage, AFP levels, lymphatic metastasis status, and differentiation degree, were recorded and summarized in Table S9. All specimens were fixed in 10% formalin, embedded in paraffin, and cut into 4 µm serial sections for immunohistochemical staining, in addition to the usual hematoxylin-eosin staining.

#### Immunohistochemistry

For immunohistochemical staining, tissues were fixed in 10% buffered formalin and embedded in paraffin. Commercially available rabbit anti-human polyclonal antibody against MAPK1 (dilution 1∶100; Catalog No.: 51068-1-AP; ProteinTech Group, Inc., Chicago, IL, USA) and rabbit anti-human polyclonal antibody against NCOA2 (dilution 1∶1000; Catalog No.: ab10508; Abcam (Hong Kong) Ltd., Hong Kong) were used. Immunohistochemical staining was carried out on sections using the avidin-biotin method and a commercially available kit (Vectastain Elite ABC kit, Vector Laboratories, Burlingame, CA). Briefly, sections (4 µm thick) were incubated overnight at 4°C with the primary antibodies against MAPK1 and NCOA2, respectively. After being washed in PBS, a biotin-marked secondary antibody was applied for 10 min followed by a peroxidase-marked streptavidin for an additional 10 min. The reaction was visualized by using 3, 3′-diaminobenzidine tetrahydrochloride. The nuclei were counterstained with hematoxylin. Negative controls were carried out by omitting the primary antibodies while MAPK1 and NCOA2 overexpression confirmed by Western blotting were used as positive controls, respectively.

Immunoreactivity was assessed by two investigators who were blinded to clinicopathologic data. Discrepancies were resolved by simultaneous reexamination of the slides by both investigators using a double-headed microscope. A semiquantitative scoring system was used as previously reported [18]. The stain was graded as 0 (negative), 1(weak), 2 (moderate), and 3 (strong). The final score was the sum total of the product of the staining intensity and corresponding tumor percentage. For example, if a tumor showed 50% moderate staining and 50% strong staining, the final score would be (50×2)+(50×3) = 250. A final score of at least 100 was considered positive expression.

### Statistical Analysis

A comparison of the prediction performance of HCC classifiers with different hub/non-hub genes was made using Fisher's exact test for any 2×2 tables by using SPSS13.0 [19]. Differences were considered to be statistically significant when the P value was less than 0.05.

## Results

### Identification of candidate HCC markers for network analysis

Data mining of three microarray datasets from the Oncomine platform for genes differentially expressed in HCC tissues compared with their expression in non-tumor liver tissues led to the identification of a list of 6582 upregulated and 5101 downregulated gene expression profiles. Then, 1098 cancer-related genes (including 469 upregulated and 629 downregulated genes) were searched from this gene list by a combination of controlled Concept filter terms. When the retrieved genes were further filtered by the corrected false discovery rate Q (Q≤0.05) and intersection screening, 116 upregulated and 111 downregulated genes were selected as candidate marker genes for further network analysis (the detailed information of this gene list was shown in Table S2). We used a stringent corrected false discovery rate cut-off value to select differentially expressed genes and to avoid false predictions arising from experimental variation in different studies.

### Identification of network hub genes for HCC classifier

**Figure 2:**
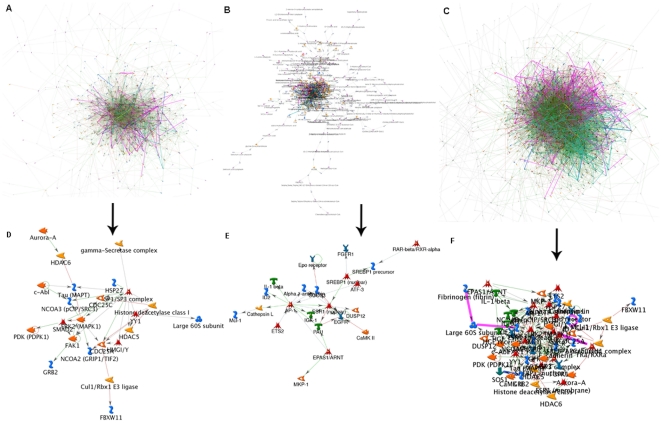
Network for upregulated genes (A), downregulated genes (B) and all differentially expressed genes (C). Hub-based network view of 10 upregulated hub genes (D), 7 downregulated hub genes (E) and 27 differentially expressed hub genes (F). GeneGO MetaCore was used to generate a network of direct connections among genes selected for analysis. Red, green, and gray arrows indicate negative, positive, and unspecified effects, respectively. Hubs were identified as having more than thirty connections and less than 50% of edges hidden within the network.

To create the network, the genes (nodes) and published literature-based connections (edges) were plotted using GeneGo-MetaCore (Figure 2A for upregulated genes, Figure 2B for downregulated genes and Figure 2C for both upregulated and downregulated genes). The network architecture is consistent with a scale-free network and represents interactions between individual targets. As the targets with high degrees of connectivity are considered to be the most important components of a network [20], we examined hubs with more than 30 connections and less than 50% of edges hidden within the network. For upregulated genes network, the 10 hubs are shown in Figure 2D: MAPK1,SP1, HDAC1, YY1, ABL1, PTK2, SMAD2, NCOA3, CDC25A, and NCOA2; for downregulated genes network, the 7 hubs are shown in Figure 2E: FOS, ESR1, JUNB, EGFR, SOCS3, FOLH1, and IGF1; for both upregulated and downregulated genes network, the 27 hubs are shown in Figure 2F: SP1, JUN, FOS, ESR1, JUNB, HDAC1, EGFR, YY1, PTK2, MAPK1, ABL1, CDH1, SMAD2, NCOA3, SOCS3, HGF, GRB2, IGF1, NCOA2, ETS2, ATF3, CDC25A, SERPINE1, DUSP1, ID2, MAPT and SREBF1 (the detailed information of these hub genes is shown in Tables S3, S4, S5).

### Performance evaluation of HCC classifier

#### Independent validation

The independent microarray gene expression datasets were used to test our HCC classifier. Chen_Liver [15] (29 HCC samples and 17 non-tumor liver tissues) and Wurmbach_Liver [16] (35 HCC samples and 27 non-tumor liver tissues) datasets were randomly separated into the training and test datasets, and this procedure was repeated 100 times. The weights of hub genes and score threshold in the HCC classifier were trained by the training dataset. The predictive accuracy and AUC value of the algorithm was then evaluated by the test datasets, and this procedure was repeated 100 times. Finally, the accuracy and AUC values for different tests were summed to calculate the average and standard error.

The overall predictive accuracy and AUC values of the different HCC classifiers, which were constructed with the 10 upregulated hub genes (Classifier 1), 7 downregulated hub genes (Classifier 2) and 27 differentially expressed hub genes (Classifier 3), on the Chen_Liver and Wurmbach_Liver test datasets were shown in Table 1, respectively. Firstly, the accuracy values of three classifiers on different independent test datasets were 85.88(92.71% and the AUC values were all more than 0.92(0.96. The AUC value is an indicator of the efficacy of the assessment system. An ideal test with perfect discrimination (100% sensitivity and 100% specificity) has an AUC of 1.0, whereas a non-informative prediction has the area 0.5, indicating that it may be achieved by mere guess. The closer to 1.0 the AUC of a test is, the higher the overall efficacy of the test will be [17]. We found that our HCC classifier had an area approximating 1.0, suggesting that it had a relatively high ability to identify the true HCC tissues against the different independent test datasets. Secondly, Classifier 3 includes more hub genes (27) than Classifier 1 (10) and Classifier 2 (7), but its performance (ACC and AUC) was not significantly higher than that of the other two classifiers. Thirdly, Classifier 2 includes the smallest number of genes and was not significantly different from other two classifiers in terms of performance, so we chose Classifier 2 for further validation.

**Table 1:** Performance of HCC classifiers on different independent test datasets.

	Wurmbach_Liver test dataset		Chen_liver test dataset	
	Acc (%)	AUC	Acc (%)	AUC
**Classifier 1**	87.20±5.72	0.92±0.04	85.88±9.70	0.94±0.05
**Classifier 2**	86.44±5.56	0.93±0.03	89.54±6.52	0.99±0.02
**Classifier 3**	88.05±4.89	0.96±0.02	92.71±7.80	0.96±0.03

#### Five-fold cross-validation

**Figure 3:**
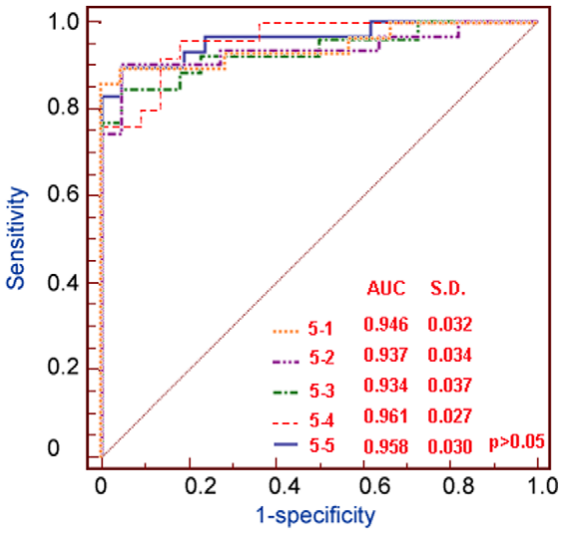
ROC curves for 5-fold cross-validations against the golden standard datasets. Each point on the ROC curve denotes the sensitivity and specificity against a set of weights and score threshold. Different colors are used to distinguish the curves of classifier in cross-validations for five times. AUC values are also presented in the figure. Sensitivity and specificity are computed during the 5-fold cross-validations (see text for details).

We also used the 5-fold cross-validation protocol to evaluate the performance of our HCC classifier (Classifier 2). The resulting ROC curves were illustrated in Figure 3. Each point on the ROC curve denotes the sensitivity and specificity against a set of weights and score threshold. Because the AUC is an indicator of the discriminatory power for the classifier, it was also used here to evaluate the predictive efficacy of our HCC classifier. From Figure 3, we can find that our HCC classifier had an AUC value approximating 1.0 in all the five tests, suggesting that it has a great reliability and efficacy to identify the true HCC tissues against different test datasets.

#### Validation of the contribution of the network topological features to the classifier

**Figure 4:**
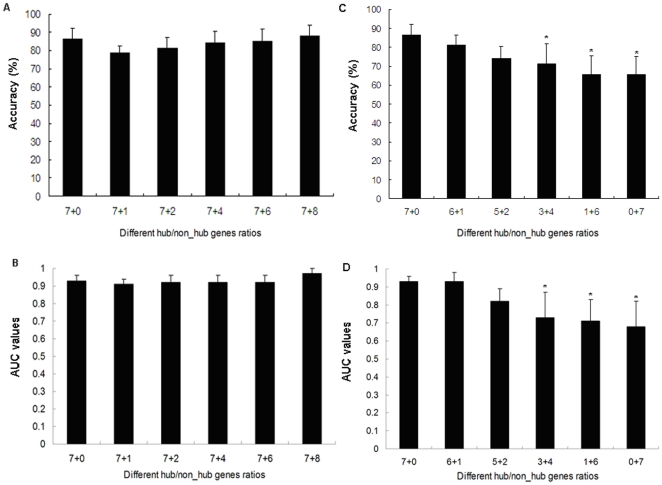
Performance of HCC classifier (Classifier 2) with adding new non-hub genes (A for predictive accuracy and B for AUC values) and with different ratios of hub and non-hub genes (C for predictive accuracy and D for AUC values). A and B showed that the predictive performance of the classifier does not change significantly with the non-hub genes being added (*p*>0.05); C and D indicated that the classifier worked much less properly with the hub genes being gradually reduced and non-hub genes gradually increased. Especially, the predictive performance of the classifier was decreased with statistic significance when the proportion of hub genes was reduced to 3/7 of the original one (*p<0.05).

In order to verify the contribution of the network topological features to the predictive performance of our classifier, we first added different proportions of non-hub genes into the classifier (Classifier 2). At each ratio (7/1, 7/2, 7/4, 7/6 and 7/8) of hub and non-hub genes, the non-hub genes were selected randomly, the process was repeated 100 times and the average performance was shown in Figure 4A∼B. From the result we can see that the predictive accuracy and AUC values of the classifier undergo no significant changes with the non-hub genes added, indicating that non-hub genes contribute little to this classifier. Then, we changed the ratio of hub and non-hub genes in the classifier (Classifier 2). At each ratio (6/1, 5/2/3/4, 1/6 and 0/7), the hub and non-hub genes were both selected randomly, the total number of them was maintained at 7. This process was repeated 100 times and the average performance was shown in Figure 4C∼D. The result shows that the classifier worked much less efficiently with the number of hub genes being gradually reduced and the number of non-hub genes gradually increased. Especially, the predictive accuracy and AUC values of the classifier were decreased with statistic significance when the proportion of hub genes was reduced to 3/7 of the original one, indicating that the network topological features integrated into modeling could improve the predictive performance of our classifier.

### Application of this modeling strategy to other cancers

In the above sections, we constructed HCC classifier by combining differential gene expression and topological characteristics of the interaction network to identify marker genes using microarray expression data of HCC and validated that this classifier has excellent predictive performance. We hypothesized that this modeling strategy could be also applied to constructing other cancers diagnosis classifiers. To test this hypothesis, we used the same method to construct the prostate cancer diagnosis classifier.

As the first step, two publicly available datasets of gene expression profiles on prostate cancer were chosen, including Liu_Prostate [21] (Normal prostate vs. prostate cancer) and Varambally_Prostate [22] (Normal prostate vs. prostate cancer). The detailed information about the datasets is described in Raw Data S6, S7, S8 and Table S6. Next, in the Oncomine platform, the genes differentially expressed in prostate cancer tissues relative to their corresponding normal tissues were filtered by a corrected Q value (Q≤0.05) cut-off and Concept filters. 21 upregulated and 27 downregulated genes that different microarray datasets of prostate cancer have in common were chosen as the candidate markers for network analysis (The gene lists were shown in Table S7). Then, their networks (Figure S1) were analyzed by GeneGO Meta-Core software and 4 hub genes were chosen, including BCL2, TP53, DAPK1 and CCND2 (The hub gene lists were shown in Table S8). After that, the prostate cancer diagnostic classifier was constructed by PLS modeling based on the microarray gene expression data of the hub genes. Furthermore, this classifier was tested by the independent microarray gene expression dataset. The overall predictive accuracy was 84.79±6.53% and AUC value was 0.82±0.10. To verify the contribution of the network topological features to the predictive performance of this prostate cancer classifier, we also changed the ratio of hub and non-hub genes in the model. As shown by the results (Figure S2), we noticed that the predictive accuracy and AUC values of this classifier were not significantly different from those of the hub genes which were not being changed and non-hub genes which were being added, but were decreased significantly when the proportion of hub genes was reduced, as was the case with the results of HCC classifier. (The detailed information on the methods and results of this section were shown in File S1.)

### Biological interpretations of hub genes in HCC classifier

Cancer diagnosis classifier does not necessarily involve understanding the biological function and regulatory mechanism of the gene components. However, molecular understanding of the biological function could still be worthwhile in that the differential expression of these genes and the interaction among them may be mechanistically linked to carcinogenesis. We therefore surveyed the literature and the knowledge databases such as Entrez Gene [23], PubMed (http://www.ncbi.nlm.nih.gov/sites/entrez?db=pubmed), and Gene References into Function (GeneRIFs) (ftp://ftp.ncbi.nih.gov/gene/GeneRIF/) on these hub genes. As a result, in the 17 (10 unregulated and 7 downregulated expression in HCC tissues) hub genes, 13 genes (SMAD2, PTK2, MAPK1, HDAC1, CDC25A, IGFI, FOS, ESR1, EGFR, SOCS3, SP1, YY1 and JUNB) have been identified as an HCC-related gene. Further studies on HCC could focus on the remaining 4 genes (ABL1, NCOA3, NCOA2 and FOLH1). In this section, we introduced the biological features of the two most promising genes.

#### EGFR

Epidermal growth factor receptor (EGFR) is a member of a proto-oncogene family of receptors important in cell proliferation [24]. The protein encoded by this gene is a transmembrane glycoprotein that is a member of the protein kinase superfamily. This protein is a receptor for members of the epidermal growth factor family. EGFR overexpression has been demonstrated in many human carcinomas including the breast, stomach, esophageal squamous carcinoma, and HCC [25]. Because of the high prevalence of EGFR overexpression in carcinomas, inhibitors of epidermal growth factor (EGF) signaling are potential therapeutic agents. In normal hepatocytes, ligand binding to EGFR results in receptor dimerization and activation of several possible pathways that transmit signals to the nucleus including STAT-1, STAT-3, STAT-5, and MAPK [26]. EGFR also signals through AKT in some cases [27]. In HCC, overexpression of EGFR has been associated with late-stage disease, increased cell proliferation, and the degree of tumor differentiation [28]. Early studies of EGFR inhibitors in HCC cell lines and phase II studies in human HCC have been encouraging.

#### PTK2

Protein tyrosine kinase 2 (PTK2) encodes a cytoplasmic protein tyrosine kinase which is found concentrated in the focal adhesions that form between cells growing in the presence of extracellular matrix constituents [29]. The encoded protein is a member of the focal adhesion kinase (FAK) subfamily of protein tyrosine kinases but lacks significant sequence similarity to kinases from other subfamilies. In HCC cell lines, the enhanced expression of FAK changed the distributions of cytoskeleton in the 3D reconstituted basement membrane and increased the adhesion and invasion potentials of cells [30]. It also has been demonstrated that SOCS-3 negatively regulates cell growth and cell motility by inhibiting Janus kinase/STAT and FAK signalings in HCC cells [31]. In HCC tissues, FAK expression is significantly related to subsequent metastasis [32]. Given the important role of FAK in tumorogenesis, metastasis and survival signaling, it is regarded as a potential target for novel anti-cancer drugs.

### Validation of MAPK1 and NCOA2 hubs

We examined the hub-based model and chose to validate the associations of MAPK1 and NCOA2 expression patterns with the clinicopathological features of HCC, respectively.

**Figure 5:**
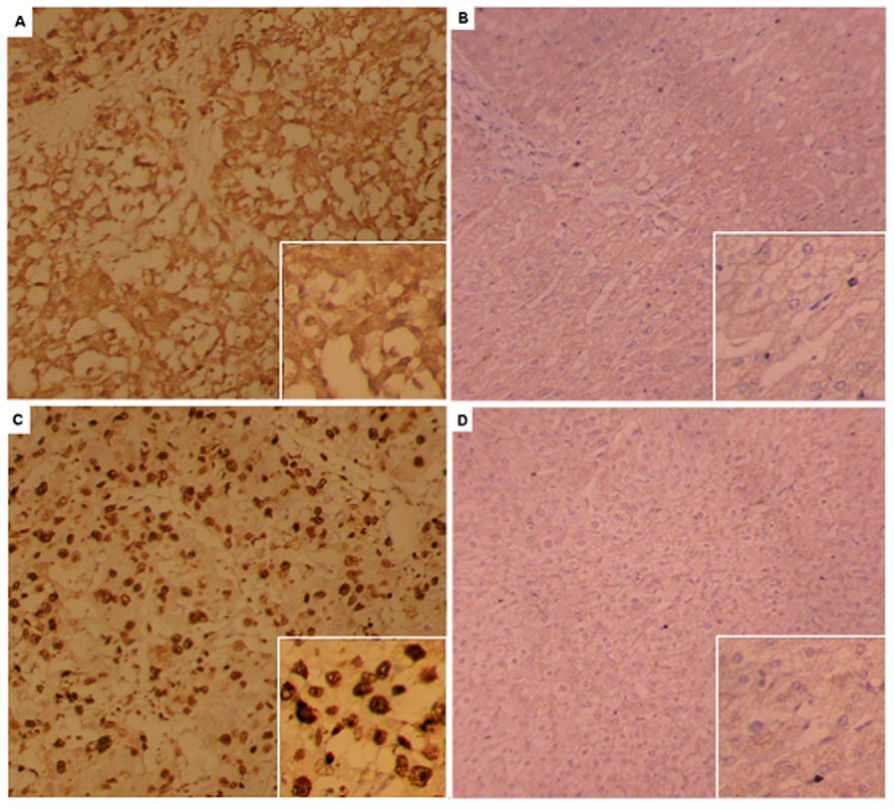
Immunohistochemical staining for MAPK1 and NCOA2 (Original magnification×200). A, MAPK1 expression was found in cell cytoplasm and/or nucleus at various levels in HCC tissues; B, MAPK1 staining was negative in paracarcinomatous liver tissues; C, NCOA2 expression was found in nucleus of tumor cells at various levels in HCC tissues; D, NCOA2 staining was negative in paracarcinomatous liver tissues.

MAPK1 was chosen because it was identified as a central hub and had the largest number of network connections. Although it has been demonstrated that the overactivation of MAPK pathway is implicated in the pathogenesis of HCC, the functional role of MAPK1 in the progression of HCC is under debate. The results of immunohistochemical staining proved that MAPK1 expression was absent or sporadic in PCLTs, whereas the distribution of HCC cells with MAPK1 immunoreactivity occurred diffusely or focally (Figure 5A and B, respectively). The MAPK1-positive cells showed unequivocal cytoplasmic and/or nuclear staining patterns. Of the 30 HCC tissues, 25 (83.33%) were positively expressed MAPK1, whereas only 7 (23.33%) of 30 PCLTs were evaluated as belonging to the MAPK1-positive group (p<0.01) (The detailed information was shown in Table S10). Then, we compared the clinicopathological findings of the MAPK1-positive and MAPK1-negative groups (Table 2). The expression patterns of MAPK1 in HCC tissues were significantly associated with the differentiation degree (p = 0.03). Tumors with positive MAPK1 expression had lower differentiation degree than those with negative expression. Although there was no statistic significance, the patients with positive MAPK1 expression tended to have higher Edmondson-Steiner grade and higher AFP level than those with negative expression. No signicant correlations were observed with other parameters, including age, gender, portal vein infiltration status, TNM stage, and lymphatic metastasis status (Table 2).

**Table 2:** Association of MAPK1 expression pattern with different clinicopathologic features of HCC.

Parameters	N	MAPK1 Expression levels (n, %)		*p*
		Negative	Positive	
**Gender**				
Male	22	3 (13.64)	19 (86.36)	0.30
Female	8	2 (25.00)	6 (75.00)	
**Age**	30	56.20±11.41	53.64±11.40	0.42
**Portal vein infiltration**				
Absence	28	5 (17.86)	23 (82.14)	0.69
Presence	2	0 (0)	2 (100.00)	
**Edmondson-Steiner grade**				
I∼II	20	5 (25.00)	15 (75.00)	0.11
III∼IV	10	0 (0)	10 (100.00)	
**TNM stage**				
I∼II	25	5 (20.00)	20 (80.00)	0.37
III∼IV	5	0 (0)	5 (100.00)	
**AFP (ng/mL)**				
≤400	22	5 (22.73)	17 (77.27)	0.18
>400	8	0 (0)	8 (100.00)	
**lymphatic metastasis**				
Absence	28	5 (17.86)	23 (82.14)	0.69
Presence	2	0 (0)	2 (100.00)	
**Differentiation degree**				
high	11	4 (36.36)	7 (63.64)	0.03
Moderate∼low	19	1 (6.25)	18 (93.75)	

NCOA2 was chosen because its correlation with HCC progression had never been reported according to our literature retrieval. From the results of immunohistochemical staining, we noticed that NCOA2 expression was absent or sporadic in PCLTs, whereas the distribution of HCC cells with NCOA2 immunoreactivity occurred diffusely or focally (Figure 5C and D, respectively). The NCOA2-positive cells showed unequivocal nuclear staining patterns. Of the 30 HCC tissues, 24 (80.00%) were positively expressed NCOA2, whereas only 4 (13.33%) of 30 PCLTs were evaluated as belonging to the NCOA2-positive group (p<0.01) (The detail information was shown in Table S11). Then, we compared the clinicopathological findings of the NCOA2-positive and NCOA2-negative groups (Table 3). The expression patterns of NCOA2 in HCC tissues were significantly associated with the Edmondson-Steiner grade (p = 0.04). The positive expression rate of NCOA2 in Edmondson-Steiner grade III∼IV group was significantly higher than that in Edmondson-Steiner grade I∼II group. Although there was no statistic significance, the patients with positive NCOA2 expression tended to have higher AFP level and lower differentiation degree than those with negative expression. No signicant correlations were observed with other parameters, including age, gender, portal vein infiltration status, TNM stage, and lymphatic metastasis status (Table 3).

**Table 3:** Association of NCOA2 expression pattern with different clinicopathologic features of HCC.

Parameters	N	NCOA2 Expression levels (n, %)		*p*
		Negative	Positive	
**Gender**				
Male	22	4 (18.18)	18 (81.82)	0.34
Female	8	2 (25.00)	6 (75.00)	
**Age**	30	55.92±11.39	52.38±11.26	0.45
**Portal vein infiltration**				
Absence	28	6 (21.43)	22 (78.57)	0.63
Presence	2	0 (0)	2 (100.00)	
**Edmondson-Steiner grade**				
I∼II	20	6 (30.00)	14 (70.00)	0.04
III∼IV	10	0 (0)	10 (100.00)	
**TNM stage**				
I∼II	25	6 (24.00)	19 (76.00)	0.30
III∼IV	5	0 (0)	5 (100.00)	
**AFP (ng/mL)**				
≤400	22	6 (27.27)	16 (72.73)	0.13
>400	8	0 (0)	8 (100.00)	
**lymphatic metastasis**				
Absence	28	6 (21.43)	22 (78.57)	0.63
Presence	2	0 (0)	2 (100.00)	
**Differentiation degree**				
high	11	4 (36.36)	7 (54.55)	0.09
Moderate∼low	19	2 (12.50)	17 (56.25)	

## Discussion

Early and accurate diagnosis of HCC is crucial to the development of patient tailored treatment strategies and the improvement of patient prognosis. In this study, we developed a novel classifier of HCC diagnosis that is based on integrating the topological features of protein-protein interaction network with gene expression data under disease conditions. The applicability of this systems biology classifier of HCC diagnosis is supported by several observations. First, we identified 10 up-regulated and 7 down-regulated hub genes that are believed to play a central role in the progression of HCC. Among these hubs, 10 are known to play a mechanistic role in the carcinogenesis of HCC and the others also have been identified as potential cancer related genes. In addition, we confirmed that the expression patterns of 2 hubs–MAPK1 and NCOA2 are significantly correlated with differentiation degree and Edmondson-Steiner grade of HCC tissues, respectively. Secondly, we developed a PLS model of HCC classification using the hub-gene systems model. This was independently validated by two test datasets from different microarray platforms, their predictive accuracy was both more than 85.00% and AUC values both approached 1.0. Thirdly, by comparing the predictive performance of our classifier with different ratios of hub and non-hub genes, we noticed that its reliability and efficacy were decreased significantly with the decline in the number of hub genes, which reflects the important contribution of topological features of network to this classifier. Furthermore, we confirmed that this modeling strategy is also applicable to the diagnosis of other cancers, such as prostate cancer.

When searching for a consensus cancer classifier, some studies have applied a combined analysis of several microarray expression datasets and used certain mathematical methods such as Logistic Discrimination, Quadratic Discriminant Analysis, or analysis of variance to “correct” systematic biases existing within those data ets to train classifiers. Scherf et al. [33] used average-linkage clustering for tumor tissues from various sites of origin. Support Vector Machine was applied to the classification of tumor and normal ovarian tissues by Furey et al [34]. While these methods are certainly a step forward in the right direction, they may bring about some problems as well. Experimental biases present in similar datasets generated in different laboratories using different microarray platforms can be possibly lessened or removed by those methods. However, if datasets contain diverse patient populations, technical and biological effects embedded in the microarray data cannot be differentiated. Thus, the application of those methods to ‘correct’ such microarray data will remove informative biological variability. To address this problem, some studies of gene networks have already been used in identification of the signature of disease mechanisms, investigation of cellular regulatory processes, hub gene analysis, and molecular characterization of the cellular state. Segal et al. [35] developed a module-network approach to the identification of modules of coregulated genes by using microarray data, enrichment analysis and promoter analysis, which is further applied to discover the signature of the mechanisms underlying tumorigenesis. Calvano et al. [36] integrated the structured network knowledge-base approach, pathway analysis and microarray data analysis to develop an analytic method of systemic inflammation. Liu et al. [37] developed a framework based on cancer class-specific gene networks for classification and pathway analysis using microarray data. Furthermore, Lu et al [10] indicated that a major challenge to the analysis of microarray data is the dependence of the interpretation of the biological relevance of changes in expression on fold change or statistics. As both approaches preferentially select genes with huge changes in expression, they found that many genes with important biological functions would not be detected. Specifically, hub genes, which are of high connectivity and putatively high biological importance, may not be detected. This is why biological understanding is enhanced by combining information including levels of change in gene expression plus topological criteria from the analysis of interaction networks.

In our classifier, the systematic integration of the differential expression analysis on microarray data and topological features of protein interaction network offers us two main advantages: First, it enables us to sufficiently utilize the gene co-expression information provided by the microarray data, which is believed to be more informative than expression changes of individual genes for target gene identification. Second, network analysis is a powerful tool to understand pathological mechanisms of disease. By integrating the topological features of biological network, some information lost in the differential expression analysis is added to our classifier.

There are, however, some flaws with our classifier. First, the analysis favored well-studied genes, because the published literature-based primary interconnections were key criteria used to build the network. We took this approach because hub connectivity is correlated with the biological importance in yeast studies [38]. Thus, the current model might not be as effective for identifying orphan genes that function as central hubs in the network. Second, although connectivity is the most important topological feature for the components of biological networks, this information is incomplete. Future studies integrating more characteristics into our classifier should be able to provide a keener insight into the network topology and tumorigenesis.

In conclusion, we developed a systems biology-based gene expression classifier of HCC diagnosis by combining information including levels of change in gene expression plus topological features from the analysis of protein interaction networks. The accuracy and stability of the predictive performance were confirmed. Our modeling strategy may also prove useful for diagnosis of other cancers.

## Supporting Information

Figure S1 **Network for upregulated genes (A), downregulated genes (B), all differentially expressed genes (C), and 4 hub genes (D).** GeneGO MetaCore was used to generate a network of direct connections among genes selected for analysis. Red, green, and gray arrows indicate negative, positive, and unspecified effects, respectively. Hubs were identified as having more than 20 connections and less than 50% of edges hidden within the network.
(TIF)

Figure S2 **Performance of prostate cancer classifier with adding new non-hub genes (A for predictive accuracy and B for AUC values) and with different ratios of hub and non-hub genes (C for predictive accuracy and D for AUC values).** A and B shown that the predictive performance of the classifier has no significant changes with the non-hub genes being added (*p*>0.05); C and D indicated that the classifier worked considerably poor with the hub genes being gradually reduced and non-hub genes gradually increased (*p<0.05).
(TIF)

Table S1 Detailed information about public expression datasets of HCC.
(DOC)

Table S2 List of 116 upregulated and 111 downregulated genes as candidate markers.
(DOC)

Table S3 Hub genes of the network of upregulated genes. Genes in blue were used as central hubs.
(DOC)

Table S4 Hub genes of the network of downregulated genes. Genes in blue were used as central hubs.
(DOC)

Table S5 Hub genes of the network of both upregulated and downregulated genes. Genes in blue were used as central hubs.
(DOC)

Table S6 Detailed information about public expression datasets of prostate cancer.
(DOC)

Table S7 List of 21 upregulated and 27 downregulated genes as candidate markers.
(DOC)

Table S8 Hub genes of the network of differentially expressed genes.
(DOC)

Table S9 Clinicopathologic features of 30 patients with hepatocellular carcinoma.
(DOC)

Table S10 MAPK1 expression pattern in tumor and paraneoplastic tissues.
(DOC)

Table S11 NCOA2 expression pattern in tumor and paraneoplastic tissues.
(DOC)

Raw Data S1 Chen liver data.
(TXT)

Raw Data S2 Part1 of Wurmbach Liver data.
(TXT)

Raw Data S3 Part2 of Wurmbach Liver data.
(TXT)

Raw Data S4 Part3 of Wurmbach Liver data.
(TXT)

Raw Data S5 Part4 of Wurmbach Liver data.
(TXT)

Raw Data S6 Part1 of Liu_prostate-E-TABM-26.processed.1.
(TXT)

Raw Data S7 Part2 of Liu_prostate-E-TABM-26.processed.1.
(TXT)

Raw Data S8 Varambally_Prostate-GSE3325.
(TXT)

File S1 (DOC)
