# Supplementary figures and images for: A Systems Biology-Based Classifier for Hepatocellular Carcinoma Diagnosis
Source: PLoS One. 2011 Jul 28;6(7):e22426. doi: 10.1371/journal.pone.0022426 (PMC3145651; doi:10.1371/journal.pone.0022426)

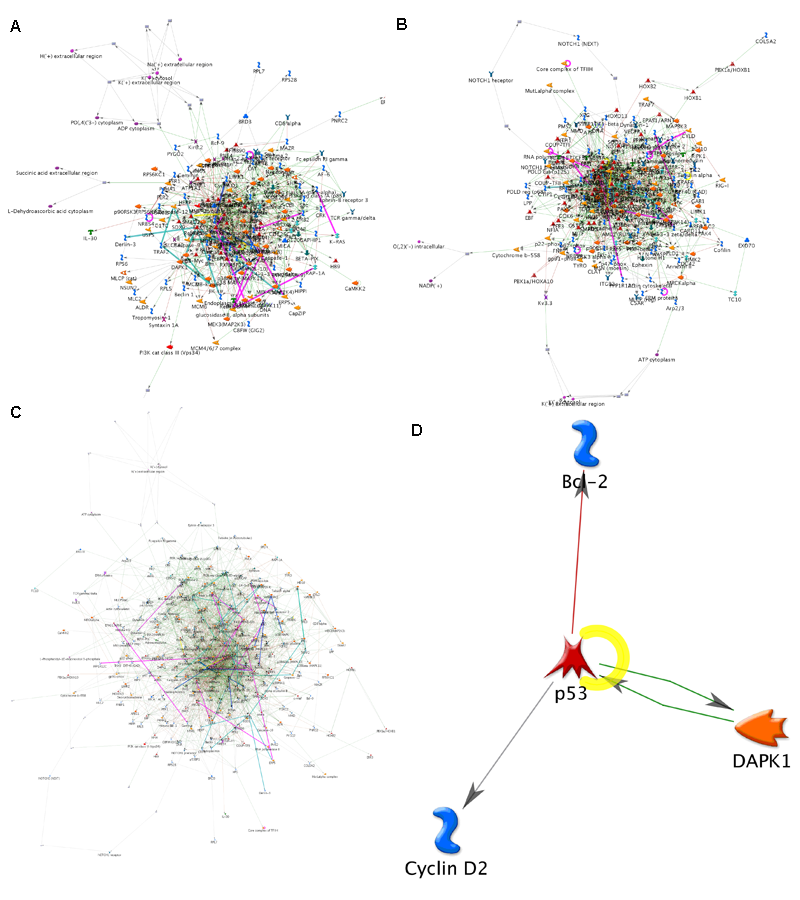

Supplement: Figure S1 — Network for upregulated genes (A), downregulated genes (B), all differentially expressed genes (C), and 4 hub genes (D). GeneGO MetaCore was used to generate a network of direct connections among genes selected for analysis. Red, green, and gray arrows indicate negative, positive, and unspecified effects, respectively. Hubs were identified as having more than 20 connections and less than 50% of edges hidden within the network. (TIF) [file pone.0022426.s001.tif]

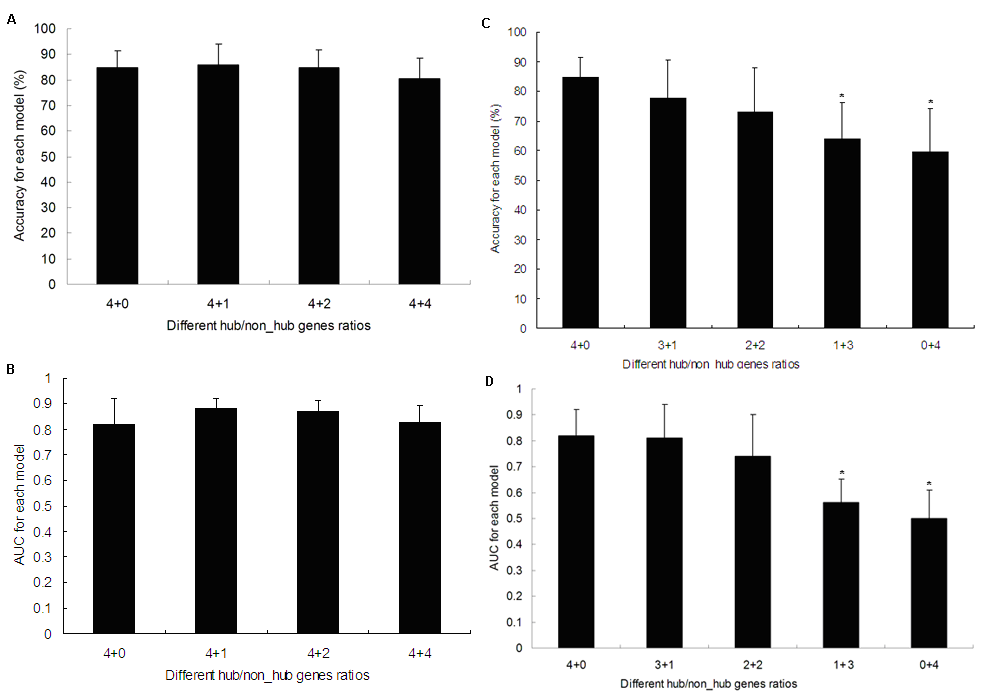

Supplement: Figure S2 — Performance of prostate cancer classifier with adding new non-hub genes (A for predictive accuracy and B for AUC values) and with different ratios of hub and non-hub genes (C for predictive accuracy and D for AUC values). A and B shown that the predictive performance of the classifier has no significant changes with the non-hub genes being added (p>0.05); C and D indicated that the classifier worked considerably poor with the hub genes being gradually reduced and non-hub genes gradually increased (*p<0.05). (TIF) [file pone.0022426.s002.tif]
