# Supplementary material for: A Systems Biology-Based Classifier for Hepatocellular Carcinoma Diagnosis
Source: PLoS One. 2011 Jul 28;6(7):e22426. doi: 10.1371/journal.pone.0022426 (PMC3145651; doi:10.1371/journal.pone.0022426)
Supplement: Table S1 — Detailed information about public expression datasets of HCC. (DOC) [file pone.0022426.s003.doc]

**Table S1 Detailed information about public expression datasets** of HCC

| **Dataset** | **Platform** | **No. of HCC samples** | **No. of non-tumor liver samples** |
| --- | --- | --- | --- |
| Wurmbach_Liver data | GPL570 | 35 | 27 |
| Chen_liver_1 data | GPL3011 | 104 | 76 |
| Chen_liver_2 data | GPL3011 | 104 | 7 |
