# Supplementary material for: A Systems Biology-Based Classifier for Hepatocellular Carcinoma Diagnosis
Source: PLoS One. 2011 Jul 28;6(7):e22426. doi: 10.1371/journal.pone.0022426 (PMC3145651; doi:10.1371/journal.pone.0022426)
Supplement: Table S2 — List of 116 upregulated and 111 downregulated genes as candidate markers. (DOC) [file pone.0022426.s004.doc]

**Table S2 List of 116 upregulated and 111 downregulated genes as candidate markers**

| **Upregulated gene symbols** | **Downregulated gene symbols** |
| --- | --- |
| ABL1 | ACADS |
| ACACA | ACADSB |
| ANAPC1 | ADK |
| ANAPC7 | ADSSL1 |
| APH1A | AGXT |
| AURKA | AGXT2 |
| BOLA2 | ALB |
| BUB1B | ALDH6A1 |
| CA12 | ALDOB |
| CAMLG | ALPL |
| CANT1 | ARG1 |
| CASK | ASL |
| CCNA2 | ASS1 |
| CCNE1 | ATF3 |
| CDC25A | BCHE |
| CDC25B | CA2 |
| CDC25C | CAMK2D |
| CHKA | CAT |
| COPS5 | CCNH |
| COX6C | COMT |
| CUTA | CTSB |
| DCK | CTSL1 |
| DDEF1 | CYP2C9 |
| DKK1 | DHRS4 |
| DNM1 | DTX1 |
| DUT | DUSP1 |
| ENAH | DUSP6 |
| EPRS | EGFR |
| EVI1 | ENPP1 |
| FBXW11 | EPAS1 |
| FNBP1 | EPHX2 |
| GGCX | EPOR |
| GM2A | ESR1 |
| GMPS | ETS2 |
| GPC3 | FAAH |
| GRB2 | FABP1 |
| GSTA4 | FDX1 |
| HDAC1 | FGA |
| HDAC5 | FGFR1 |
| HEY1 | FGFR2 |
| HGS | FHL1 |
| HMGA1 | FOLH1 |
| HNRNPA2B1 | FOS |
| HSPB1 | FVT1 |
| IRAK1 | FZD8 |
| KIF11 | GAS1 |
| KPNB1 | GPT |
| LCMT1 | HBG1 |
| LCN2 | HCK |
| LPP | HDAC6 |
| MAML1 | HEY2 |
| MAP1B | HGF |
| MAP3K7 | HIPK3 |
| MAPK1 | HSD17B10 |
| MAPT | ID2 |
| MCM5 | ID4 |
| MMP11 | IGF1 |
| MMP12 | IL1B |
| MSI2 | IL1R1 |
| MTIF2 | IL1RAP |
| MTR | IL1RN |
| MYST2 | IL4R |
| NBN | IL6ST |
| NCKIPSD | IVD |
| NCOA2 | JUNB |
| NCOA3 | KYNU |
| NCSTN | LAMA2 |
| NKIRAS2 | LIFR |
| NLK | MAP2K1 |
| NOTCH3 | MAP2K3 |
| NRBP1 | MASP2 |
| NSD1 | MBL2 |
| NUP214 | MCC |
| OGT | MCL1 |
| PARD3 | MRC1 |
| PDPK1 | MTHFD1 |
| POU2F1 | MVK |
| PRCC | MYD88 |
| PRKD2 | NFKBIZ |
| PSEN2 | NR0B2 |
| PSPH | NR3C2 |
| PTK2 | OAT |
| PURA | PDE4DIP |
| RALBP1 | PLD1 |
| RECQL4 | PLG |
| RFXANK | PPP2R2A |
| RIT1 | PTGS2 |
| RNF4 | PTPN3 |
| RPL30 | RABEP1 |
| RRM1 | RARB |
| SET | RBP1 |
| SGPL1 | RBP5 |
| SMAD2 | RCHY1 |
| SMURF1 | SDS |
| SNRPD2 | SERPINA1 |
| SNRPE | SERPINE1 |
| SOS1 | SFRP1 |
| SP1 | SGK |
| SQLE | SHBG |
| SUMO2 | SLC16A2 |
| TLX1 | SLC16A4 |
| TOP2A | SLC7A2 |
| TPI1 | SOCS3 |
| TPR | SPRY2 |
| TRAF1 | SRD5A1 |
| TRAF5 | SREBF1 |
| TRIM24 | SS18 |
| TRIM33 | THRA |
| TTL | TTR |
| TXNRD1 | XDH |
| UCK2 | YES1 |
| UNG |  |
| WDR68 |  |
| WHSC1 |  |
| WNK1 |  |
| YY1 |  |
