# Supplementary material for: A Systems Biology-Based Classifier for Hepatocellular Carcinoma Diagnosis
Source: PLoS One. 2011 Jul 28;6(7):e22426. doi: 10.1371/journal.pone.0022426 (PMC3145651; doi:10.1371/journal.pone.0022426)
Supplement: Table S3 — Hub genes of the network of upregulated genes. Genes in blue were used as central hubs. (DOC) [file pone.0022426.s005.doc]

**Table S3. Hub genes of the network of upregulated genes. Genes in blue were used as central hubs.**

| **Gene_symbol** | **Network_object** | **All_edges** | **Hidden_edges** |
| --- | --- | --- | --- |
| MAPK1 | ERK1/2 | 181 | 0 |
| SP1 | SP1 | 85 | 0 |
| HDAC1 | HDAC1 | 71 | 0 |
| YY1 | YY1 | 49 | 0 |
| ABL1 | c-Abl | 41 | 0 |
| PTK2 | FAK1 | 36 | 0 |
| SMAD2 | SMAD2 | 35 | 0 |
| NCOA3 | NCOA3-(pCIP/SRC3) | 33 | 0 |
| CDC25A | CDC25A | 32 | 0 |
| NCOA2 | NCOA2-(GRIP1/TIF2) | 31 | 0 |
| HDAC5 | HDAC5 | 23 | 0 |
| GRB2 | GRB2 | 22 | 0 |
| COPS5 | JAB1 | 20 | 0 |
| HMGA1 | HMGI/Y | 20 | 0 |
| HSPB1 | HSP27 | 20 | 0 |
| PDPK1 | PDK-(PDPK1) | 19 | 0 |
| AURKA | Aurora-A | 18 | 0 |
| FBXW11 | Cul1/Rbx1-E3-ligase | 18 | 0 |
| MAPT | Tau-(MAPT) | 18 | 0 |
| HEY1 | HEY1 | 17 | 0 |
| RPL30 | Large-60S-subunit | 17 | 0 |
| PSEN2 | Presenilin-2 | 16 | 0 |
| CDC25C | CDC25C | 15 | 0 |
