# Supplementary material for: A Systems Biology-Based Classifier for Hepatocellular Carcinoma Diagnosis
Source: PLoS One. 2011 Jul 28;6(7):e22426. doi: 10.1371/journal.pone.0022426 (PMC3145651; doi:10.1371/journal.pone.0022426)
Supplement: Table S4 — Hub genes of the network of downregulated genes. Genes in blue were used as central hubs. (DOC) [file pone.0022426.s006.doc]

**Table S4. Hub genes of the network of downregulated genes. Genes in blue were used as central hubs.**

| **Gene_symbol** | **Network_object** | **ALL_edge** | **Hidden_edge** |
| --- | --- | --- | --- |
| FOS | AP-1,JunD/c-Fos,c-Fos | 154 | 0 |
| ESR1 | ESR | 115 | 0 |
| JUNB | AP-1,FosB/JunB,JunB,JunB/Fra-1 | 112 | 0 |
| EGFR | EGFR | 75 | 0 |
| SOCS3 | SOCS3 | 36 | 0 |
| FOLH1 | FOLH1(GCP2),IL-6,SOS | 33 | 0 |
| IGF1 | IGF-1,RNASEH2A | 31 | 0 |
| DUSP1 | MKP-1,PRKCSH | 28 | 0 |
| ETS2 | ETS | 27 | 0 |
| SERPINE1 | PAI1,RBP1 | 26 | 0 |
| SREBF1 | SREBP1(Golgi membrane) | 23 | 0 |
| MCL1 | Mcl-1 | 23 | 0 |
| EPOR | Epo-receptor | 23 | 0 |
| ATF3 | ATF-3 | 23 | 0 |
| FGFR1 | FGFR1 | 22 | 0 |
| SGK | SGK1 | 21 | 0 |
| ID2 | ID2 | 21 | 0 |
| IL1B | IL-1-beta | 20 | 0 |
| RARB | RAR | 19 | 0 |
| SERPINA1 | Alpha-1-antitrypsin,TINAGL1 | 17 | 0 |
| MYD88 | MyD88 | 17 | 0 |
| EPAS1 | EPAS1 | 17 | 0 |
| CAMK2D | CaMK-II | 16 | 0 |
| CTSL1 | Cathepsin-L | 14 | 0 |
