# Supplementary material for: A Systems Biology-Based Classifier for Hepatocellular Carcinoma Diagnosis
Source: PLoS One. 2011 Jul 28;6(7):e22426. doi: 10.1371/journal.pone.0022426 (PMC3145651; doi:10.1371/journal.pone.0022426)
Supplement: Table S5 — Hub genes of the network of both upregulated and downregulated genes. Genes in blue were used as central hubs. (DOC) [file pone.0022426.s007.doc]

Table S5. Hub genes of the network of both upregulated and downregulated genes. Genes in blue were used as central hubs.

| Gene_symbol | Network_object | All-edge | Hidden_edge |
| --- | --- | --- | --- |
| SP1 | SP1 | 302 | 0 |
| JUN | AP-1 | 258 | 0 |
| FOS | AP-1 | 218 | 0 |
| ESR1 | ESR1(nuclear) | 172 | 0 |
| JUNB | AP-1 | 160 | 0 |
| HDAC1 | HDAC1 | 97 | 0 |
| EGFR | EGFR | 91 | 0 |
| YY1 | YY1 | 68 | 0 |
| PTK2 | FAK1 | 63 | 0 |
| MAPK1 | ERK1/2 | 61 | 0 |
| ABL1 | c-Abl | 60 | 0 |
| CDH1 | APC/hCDH1-complex | 57 | 0 |
| SMAD2 | SMAD2 | 43 | 0 |
| NCOA3 | NCOA3(pCIP/SRC3) | 42 | 0 |
| SOCS3 | SOCS3 | 42 | 0 |
| HGF | IL-6 | 41 | 0 |
| GRB2 | GRB2 | 40 | 0 |
| IGF1 | IGF-1 | 39 | 0 |
| NCOA2 | NCOA2(GRIP1/TIF2) | 38 | 0 |
| ETS2 | ETS2 | 37 | 0 |
| ATF3 | ATF-3 | 36 | 0 |
| CDC25A | CDC25A | 35 | 0 |
| SERPINE1 | PAI1 | 35 | 0 |
| DUSP1 | MKP-1 | 34 | 0 |
| ID2 | ID2 | 31 | 0 |
| MAPT | Tau(MAPT) | 31 | 0 |
| SREBF1 | SREBP1(nuclear) | 31 | 0 |
| IL1B | IL-1-beta | 30 | 0 |
| MCL1 | Mcl-1 | 29 | 0 |
| SGK | SGK1 | 28 | 0 |
| CTSL1 | Cathepsin-L | 27 | 0 |
| FGA | Fibrinogen(fibrin) | 27 | 0 |
| PDPK1 | PDK(PDPK1) | 27 | 0 |
| EPOR | Epo-receptor | 26 | 0 |
| FGFR1 | FGFR1 | 26 | 0 |
| RPL30 | Large-60S-subunit | 26 | 0 |
| HDAC5 | HDAC5 | 25 | 0 |
| HSPB1 | HSP27 | 25 | 0 |
| NR0B2 | SHP | 25 | 0 |
| HMGA1 | HMGI/Y | 24 | 0 |
| AURKA | Aurora-A | 23 | 0 |
| EPAS1 | EPAS1 | 22 | 0 |
| SERPINA1 | Alpha1-antitrypsin | 22 | 0 |
| MYD88 | MyD88 | 21 | 0 |
| CAMK2D | CaMK-II | 20 | 0 |
| FBXW11 | Cul1/Rbx1-E3-ligase | 20 | 0 |
| NR3C2 | MCR | 20 | 0 |
| THRA | TR-alpha | 20 | 0 |
