# Supplementary material for: A Systems Biology-Based Classifier for Hepatocellular Carcinoma Diagnosis
Source: PLoS One. 2011 Jul 28;6(7):e22426. doi: 10.1371/journal.pone.0022426 (PMC3145651; doi:10.1371/journal.pone.0022426)
Supplement: Table S6 — Detailed information about public expression datasets of prostate cancer. (DOC) [file pone.0022426.s008.doc]

**Table S6 Detailed information about public expression datasets** of prostate cancer

| **Dataset** | **Platform** | **No. of HCC samples** | **No. of non-tumor liver samples** |
| --- | --- | --- | --- |
| Liu Prostate | GPL570 | 42 | 10 |
| Varambally Prostate | GPL570 | 13 | 6 |
