# Supplementary material for: A Systems Biology-Based Classifier for Hepatocellular Carcinoma Diagnosis
Source: PLoS One. 2011 Jul 28;6(7):e22426. doi: 10.1371/journal.pone.0022426 (PMC3145651; doi:10.1371/journal.pone.0022426)
Supplement: Table S7 — List of 21 upregulated and 27 downregulated genes as candidate markers. (DOC) [file pone.0022426.s009.doc]

**Table S7 List of 21 upregulated and 27 downregulated genes as candidate markers**

| **Upregulated gene symbols** | **Downregulated gene symbols** |
| --- | --- |
| ADRBK1 | BCL11A |
| BCL9 | BCL2 |
| BRD3 | CCNB1IP1 |
| CAMKK2 | CCND2 |
| DAPK1 | CDC42BPA |
| EPHB3 | CIITA |
| ERG | CYLD |
| FGFR1OP | EPAS1 |
| HIP1 | ERCC5 |
| MLF1 | EVI1 |
| MNX1 | EXT1 |
| NONO | FGFR2 |
| NPM1 | FNBP1 |
| PATZ1 | FUS |
| RPS6KC1 | HOXD11 |
| SMOX | HOXD13 |
| SNRPE | LPP |
| TAOK3 | MAF |
| TP53 | MAST4 |
| TRIB1 | MLL |
| TTK | PBX1 |
|  | PMS2 |
|  | PRKCB1 |
|  | SPEG |
|  | TCF7L1 |
|  | TFE3 |
|  | TOX4 |
