# Supplementary material for: A Systems Biology-Based Classifier for Hepatocellular Carcinoma Diagnosis
Source: PLoS One. 2011 Jul 28;6(7):e22426. doi: 10.1371/journal.pone.0022426 (PMC3145651; doi:10.1371/journal.pone.0022426)
Supplement: Table S8 — Hub genes of the network of differentially expressed genes. (DOC) [file pone.0022426.s010.doc]

**Tables S8. Hub genes of the network** of differentially expressed genes

| **Genesymbol** | **NETwork-object** | **all_edge** | **hidden_edge** |
| --- | --- | --- | --- |
| TP53 | p53 | 60 | 0 |
| BCL2 | Bcl-2 | 30 | 0 |
| CCND2 | Cyclin | 30 | 0 |
| DAPK1 | DAPK1 | 26 | 0 |
