# Supplementary material for: A Systems Biology-Based Classifier for Hepatocellular Carcinoma Diagnosis
Source: PLoS One. 2011 Jul 28;6(7):e22426. doi: 10.1371/journal.pone.0022426 (PMC3145651; doi:10.1371/journal.pone.0022426)
Supplement: Table S9 — Clinicopathologic features of 30 patients with hepatocellular carcinoma. (DOC) [file pone.0022426.s011.doc]

**Table S9 Clinicopathologic features of 30 patients with hepatocellular carcinoma**

| **Parameters** | **N** |
| --- | --- |
| Mean age (years, Mean±S.D.) | 52.4±11.1 |
| Sex (male/female) | 22/8 |
| Etiological factors |  |
| HBV(+)/HBV(-) | 23/7 |
| Underlying disease |  |
| Chronic hepatitis/cirrhosis | 22/24 |
| Portal vein infiltration |  |
| Absence / Presence (a1/a2) | 28/2 |
| Edmondson-Steiner grade |  |
| I~II/ III~IV (b1/b2) | 20/10 |
| TNM stage |  |
| I~II/ III~IV (c1/c2) | 25/5 |
| AFP (ng/mL) |  |
| ≤400/>400 (d1/d2) | 22/8 |
| lymphatic metastasis |  |
| Absence / Presence (e1/e2) | 28/2 |
| Differentiation degree |  |
| high/moderate/low (f1/f2/f3) | 11/16/3 |
| Total | 30 |
