# Supplementary material for: A Systems Biology-Based Classifier for Hepatocellular Carcinoma Diagnosis
Source: PLoS One. 2011 Jul 28;6(7):e22426. doi: 10.1371/journal.pone.0022426 (PMC3145651; doi:10.1371/journal.pone.0022426)
Supplement: Table S10 — MAPK1 expression pattern in tumor and paraneoplastic tissues. (DOC) [file pone.0022426.s012.doc]

Table S10 MAPK1 expression pattern in tumor and paraneoplastic tissues

| HCC tissues (n, %) | | | PCLTs | | | *P* |
| --- | --- | --- | --- | --- | --- | --- |
| 0 | 1 | 2 | 0 | 1 | 2 |
| 5 (16.67) | 18 (60.00) | 7 (23.33) | 23 (76.67) | 7 (23.33) | 0 (0) | <0.01 |

Note: ‘0’, ‘1’ and ‘2’ refer to negative, weak positive and strong positive expression, respectively.
