# Supplementary material for: A Systems Biology-Based Classifier for Hepatocellular Carcinoma Diagnosis
Source: PLoS One. 2011 Jul 28;6(7):e22426. doi: 10.1371/journal.pone.0022426 (PMC3145651; doi:10.1371/journal.pone.0022426)
Supplement: Table S11 — NCOA2 expression pattern in tumor and paraneoplastic tissues. (DOC) [file pone.0022426.s013.doc]

**Table S11 NCOA2** expression pattern in tumor and paraneoplastic tissues

| HCC tissues (n, %) | | | PCLTs | | | *P* |
| --- | --- | --- | --- | --- | --- | --- |
| 0 | 1 | 2 | 0 | 1 | 2 |
| 6 (20.00) | 16 (53.33) | 8 (26.67) | 26 (86.67) | 4 (13.33) | 0 (0) | <0.01 |

Note: ‘0’, ‘1’ and ‘2’ refer to negative, weak positive and strong positive expression, respectively.
