# Supplementary material for: A Systems Biology-Based Classifier for Hepatocellular Carcinoma Diagnosis
Source: PLoS One. 2011 Jul 28;6(7):e22426. doi: 10.1371/journal.pone.0022426 (PMC3145651; doi:10.1371/journal.pone.0022426)
Supplement: File S1 — (DOC) [file pone.0022426.s022.doc]

**Supplemental Data**

**for**

**A System Biology-Based Gene Expression Classifier of Cancer Diagnosis**

***Systems biology-based prostate cancer classifier construction***

*Gene selection procedure*

Prostate cancer was used in the <profile search> function in the Oncomine database to find the available microarray datasets related to the specific cancer type. The analysis type <cancer vs. normal> was then applied to filter those microarray datasets exploring cancer relative to its normal tissue. Two publicly available datasets of gene expression profiles were chosen, including Liu_Prostate [1] (Normal prostate vs. prostate cancer) and Varambally_Prostate [2] (Normal prostate vs. prostate cancer). The detailed information about the datasets is described in Table S6.

Concept filters in the Oncomine database were used to identify known oncogenes differentially expressed in prostate cancer. Specifically, differentially expressed genes associated with the following Concept filter terms were searched: <CAN Genes>, <The Cancer Gene Census>, <Catalogue of Somatic Mutations in Cancer (COSMIC)>, <Drug target-experimental> and <Drug target-FDA>. Next, a corrected false discovery rate Q value threshold (Q≤0.05) was used to filter and retrieve those differentially expressed genes with a high confidence. Then, 21 upregulated and 27 downregulated genes that are in common among different microarray datasets of prostate cancer were chosen as the candidate markers for network analysis (The gene lists were shown in Supplementary Table S7).

*Identification of network hub genes for prostate cancer classifier*

To create the network, the genes (nodes) and published literature-based connections (edges) were plotted using GeneGo-MetaCore (Figure S1A for upregulated genes, Figure S1B for downregulated genes and Figure S1C for both upregulated and downregulated genes). The network architecture is consistent with a scale-free network and represents interactions between individual targets. Because the targets with high degrees of connectivity are considered to be the most important components of a network [3], we examined hubs with more than 20 connections and less than 50% of edges hidden within the network. The 4-hub network was shown in Figure S1D: BCL2, TP53, DAPK1 and CCND2 (the detail information of these hub genes was shown in Tables S8).

*Performance evaluation of prostate cancer classifier*

PLS was used to construct this systems biology-based prostate cancer classifier, with the same method as HCC classifier.

The independent microarray gene expression datasets was used to test our prostate cancer classifier. Liu_Prostate dataset was randomly separated into the training and test datasets for 100 times. The weights of hub genes and score threshold in the classifier were trained by the training dataset; the predictive accuracy and AUC value of the algorithm was then evaluated by the test datasets, and this procedure was repeated 100 times, and finally the accuracy and AUC values for different tests were summed to calculate the average and standard error. The overall predictive accuracy was 84.79±6.53% and AUC value was 0.82±0.10.

In order to verify the contribution of the network topological features to the predictive performance of our classifier, we first added the different proportions of non-hub genes into the classifier. At each ratio, the non-hub genes were selected randomly, the process was repeated 100 times and the average performance was shown in Figure S2A~B. From the result we can see that the predictive accuracy and AUC values of the classifier have no significant changes with the non-hub genes being added, indicating that non-hub genes contribute little to the classifier. Then, we changed the ratio of hub and non-hub genes in the classifier. At each ratio, the hub and non-hub genes were both selected randomly, the total number of them was maintain 4, the process was repeated 100 times and the average performance was shown in Figure S2C~D. From the result we can see that the classifier worked considerably poor with the hub genes being gradually reduced and non-hub genes gradually increased, indicating that the network topological features integrated into the model could improve the predictive performance of our classifier.

**Reference**

1. Liu P, Ramachandran S, Ali Seyed M, Scharer CD, Laycock N, Dalton WB, Williams H, Karanam S, Datta MW, Jaye DL, Moreno CS. Sex-determining region Y box 4 is a transforming oncogene in human prostate cancer cells. Cancer Res. 2006; 66: 4011-4019.
2. Varambally S, Yu J, Laxman B, Rhodes DR, Mehra R, Tomlins SA, Shah RB, Chandran U, Monzon FA, Becich MJ, Wei JT, Pienta KJ, Ghosh D, Rubin MA, Chinnaiyan AM. Integrative genomic and proteomic analysis of prostate cancer reveals signatures of metastatic progression. Cancer Cell. 2005; 8: 393-406.
3. Jeong H, Mason SP, Barabasi AL, et al. Lethality and centrality in protein networks. Nature 2001; 411: 41–42.
